# Discordance of Dopaminergic Dysfunction and Subcortical Atrophy by α‐Synuclein Status in Sporadic and Genetic Parkinson's Disease

**DOI:** 10.1002/mds.70186

**Published:** 2026-01-28

**Authors:** Michael Tran Duong, Sandhitsu R. Das, Pulkit Khandelwal, Joaquin A. Vizcarra, Yue Li, Long Xie, Paul A. Yushkevich, Leslie M. Shaw, Jacob G. Dubroff, Parkinson's Progression Markers Initiative, Kenneth Marek, Shirley Lasch, Caroline Tanner, Tanya Simuni, Christopher Coffey, Karl Kieburtz, Renee Wilson, Brit Mollenhauer, Site Investigator, Douglas Galasko, Site Investigator, Tatiana Foroud, Lana Chahine, Andrew Siderowf, John Seibyl, Arthur Toga, Andrew Singleton, Daniel Weintraub, John Trojanowski, Leslie Shaw, Duygu Tosun‐Turgut, Kathleen Poston, Susan Bressman, Kalpana M. Merchant, Werner Poewe, Todd Sherer, Sohini Chowdhury, Mark Frasier, Catherine Kopil, Anna Naito, Vanessa Arnedo, Ray Dorsey, Cynthia Casaceli, Imaging Core, Nichole Daegele, Justin Albani1 Statistics Core, Chelsea Caspell‐Garcia, Liz Uribe, Eric Foster, Jeff Long, Nick Seedorff, Karen Crawford, Danielle Elise Smith, Paola Casalin, Giulia Malferrari, Cheryl Halter, David Russell, Stewart Factor, Penelope Hogarth, David Standaert, Amy Amara, Robert Hauser, Joseph Jankovic, Matthew Stern, Shu‐Ching Hu, Gretchen Todd, Rachel Saunders‐Pullman, Irene Richard, Marie H Saint‐Hilaire, Klaus Seppi, Holly Shill, Hubert Fernandez, Claudia Trenkwalder, Wolfgang Oertel, Daniela Berg, Kathrin Brockman, Isabel Wurster, Liana Rosenthal, Yen Tai, Nicola Pavese, Paolo Barone, Stuart Isaacson, Alberto Espay, Dominic Rowe, Melanie Brandabur, James Tetrud, Grace Liang, Alex Iranzo, Eduardo Tolosa, Karen Marder, Maria de Arriba Sanchez, Leonidis Stefanis, Maria Jose Marti, Javier Ruiz Martinez, Jean‐Christophe Corvol, Jan O Assly, Salima Brillman, Nir Giladi, Debra Smejdir, Julia Pelaggi, Farah Kausar, Linda Rees, Barbara Sommerfield, Madeline Cresswell, Courtney Blair, Karen Williams, Grace Zimmerman, Stephanie Guthrie, Ashlee Rawlins, Leigh Donharl, Christine Hunter, Baochan Tran, Abigail Darin, Carly Linder, Marne Baca, Heli Venkov, Cathi‐Ann Thomas, Raymond James, Beatrice Heim, Paul Deritis, Fabienne Sprenger, Deborah Raymond, Diana Willeke, Zoran Obradov, Jennifer Mule, Nancy Monahan, Katharina Gauss, Deborah Fontaine, Daniel Szpak, Arita McCoy, Becky Dunlop, Laura Marie Payne, Susan Ainscough, Lisbeth Carvajal, Rebecca Silverstein, Kristy Espay, Madelaine Ranola, Elisabet Mondragon Rezola, Helen Mejia Santana, Maria Stamelou, Alicia Garrido, Stephanie Carvalho, Anne Grete Kristiansen, Krista Specketer, Anat Mirlman, Maurizio Facheris, Holly Soares, Mark A. Mintun, Jesse Cedarbaum, Peggy Taylor, Danna Jennings, Lawrence Slieker, Brian McBride, Colin Watson, Etienne Montagut, Zulfiqar Haider Sheikh, Baris Bingol, Remi Forrat, Pablo Sardi, Tanya Fischer, Alastair D. Reith, Jan Egebjerg, Lone Frydelund Larsen, Nathalie Breysse, Didier Meulien, Barbara Saba, Vera Kiyasova, Chris Min, Thomas McAvoy, Robert Umek, Philip Iredale, Jeremy Edgerton, Susan De Santi, Christian Czech, Frank Boess, Jeffrey Sevigny, Thomas Kremer, Igor Grachev, Kaplana Merchant, Andreja Avbersek, Pierandrea Muglia, Alexandra Stewart, Rene Prashad, Johannes Taucher, Andrew Siderowf, David A. Wolk, Ilya M. Nasrallah

**Affiliations:** ^1^ Department of Radiology, Perelman School of Medicine University of Pennsylvania Philadelphia Pennsylvania USA; ^2^ Department of Neurology, Perelman School of Medicine University of Pennsylvania Philadelphia Pennsylvania USA; ^3^ Departments of Medicine, Neurology and Psychiatry, Alzheimer's Disease Research Center, Perelman School of Medicine University of Pennsylvania Philadelphia Pennsylvania USA; ^4^ Department of Pathology and Laboratory Medicine, Perelman School of Medicine University of Pennsylvania Philadelphia Pennsylvania USA

**Keywords:** α‐synuclein, DAT, MRI, Parkinson's disease, SAA

## Abstract

**Background:**

Parkinson's disease (PD) is characterized by predominantly neuronal α‐synuclein pathology and dopaminergic dysfunction. Cerebrospinal fluid (CSF) seeding amplification assays (SAA) detect α‐synuclein aggregates in vivo, but not all patients with PD have a positive SAA. This pathological heterogeneity among patients may not be entirely captured by binary results from α‐synuclein SAA positivity (S+) versus negativity (S–). To further dissect this biological variability, we explored spatial neuroimaging differences in S+ versus S– patients.

**Objective:**

The study aim was to investigate how SAA status influences imaging measures of dopamine denervation and atrophy.

**Methods:**

We compare SAA status with CSF proteinopathy markers, ^123^I‐Ioflupane dopamine transporter (DAT), and magnetic resonance imaging (MRI) in participants with sporadic (n = 490), *LRRK2*‐associated (n = 158), and *GBA*‐associated (n = 80) PD from the Parkinson's Progression Markers Initiative (PPMI).

**Results:**

Between 64% and 95% of participants in these groups have S+ status. For all groups, S+ participants have decreased putamen DAT neurotransmission compared to S– participants, whereas S– participants have reduced MRI volume in basal ganglia structures relative to S+ participants. With striatal DAT/MRI ratios, S+ participants have disproportionately lower putamen DAT uptake relative to atrophy. In exploratory analyses, participants with cognitive impairment or hyposmia are associated with worse DAT/MRI discordance. By CSF markers, S– participants with sporadic PD have higher CSF pTau_181_/amyloid‐β_42_ ratio, suggesting Alzheimer's copathology.

**Conclusions:**

S+ patients exhibit more dopaminergic deficit, whereas S– patients have more subcortical atrophy across sporadic and genetic PD. Together, our findings reveal structure/function and DAT/MRI discordance, providing insight into biomarkers and pathophysiology of synucleinopathy and PD. © 2026 The Author(s). *Movement Disorders* published by Wiley Periodicals LLC on behalf of International Parkinson and Movement Disorder Society.

Clinical heterogeneity presents substantial challenges to early, precise diagnosis of neurodegenerative disorders and motivates further investigation into disease biomarkers. Neuronal α‐synuclein inclusions form Lewy bodies and neurites^1^ and are detected by in vivo cerebrospinal fluid (CSF) tests in about 90% of patients diagnosed with sporadic Lewy body disease (LBD), which includes Parkinson's disease (PD) and dementia with Lewy bodies (DLB),^2^, ^3^, ^4^, ^5^, ^6^, ^7^, ^8^ though glial α‐synuclein inclusions are increasingly recognized in LBD as well. Recently, biological classification and staging systems^9^, ^10^ have been proposed for LBD, requiring the presence of misfolded neuronal α‐synuclein. Seeding amplification assays (SAA) can detect α‐synuclein aggregates from CSF with high sensitivity and specificity in patients with sporadic and genetic PD in the Parkinson's Progression Markers Initiative (PPMI)^7^, ^8^, ^11^ and additional α‐synucleinopathy cohorts of PD, DLB, multiple system atrophy (MSA), pure autonomic failure, rapid eye movement (REM) sleep behavior disorder, and older adults.^2^, ^3^, ^4^, ^5^, ^6^, ^12^, ^13^, ^14^, ^15^

Nonetheless, substantial variability remains in α‐synuclein pathology and CSF SAA status across myriad forms of parkinsonism.^11^, ^13^, ^16^, ^17^, ^18^ PD can be sporadic or genetic, with the most common PD‐associated genetic variants mapped to *LRRK2* and *GBA*. Although almost all patients with sporadic PD and PD associated with a *GBA* variant have positive α‐synuclein SAA, between 30% and 40% of patients with PD associated with a *LRRK2* variant have negative CSF α‐synuclein SAA.^11^ Patients with *LRRK2* and *GBA* PD may also differ in clinical presentation, including the degree of hyposmia.^7^, ^11^ Furthermore, SAA accuracy depends on α‐synuclein load and distribution, with lower SAA sensitivity in detecting α‐synuclein involvement of amygdala and brainstem at early stages compared to later stages of widespread neocortical distribution.^5^, ^19^, ^20^ Another major PD biomarker is ^123^I‐Ioflupane dopamine transporter (DAT) single‐photon emission computed tomography (SPECT), the clinical standard to evaluate striatal dopamine deficiency.^21^ Prior studies demonstrated reduction in DAT uptake for sporadic PD^22^, ^23^, ^24^, ^25^, ^26^ and genetic PD,^11^, ^27^, ^28^ but further comparisons are warranted between structural and molecular imaging measures across different α‐synuclein SAA statuses. Although pathophysiologic and spatiotemporal heterogeneity complicates the interpretation of SAA, it also inspires further inquiry into regional imaging differences between α‐synuclein SAA positive (S+) and negative (S–) patients.

It has long been recognized in LBD that molecular neurotransmission changes (particularly of dopaminergic circuits^22^, ^23^, ^24^, ^25^, ^26^, ^27^, ^28^) predominate over gross volume loss, in contrast to other neurodegenerative disorders.^10^, ^29^, ^30^, ^31^ The present investigation is motivated by previous literature that highlighted a spatial mismatch between imaging modalities^32^, ^33^ in mixed Alzheimer's disease (AD) and a dissociation between structural atrophy and ^18^F‐Fluorodeoxyglucose (^18^F‐FDG) positron emission tomography (PET) in α‐synucleinopathy.^34^ Molecular ^18^F‐FDG PET is commonly used to distinguish between neurodegenerative disorders, especially DLB,^20^, ^35^, ^36^ where metabolic changes are often greater than the degree of corresponding atrophy.^37^, ^38^, ^39^, ^40^ In fact, patients with mixed DLB + AD display disproportionately more parieto‐occipital ^18^F‐FDG hypometabolism relative to cortical atrophy or ^18^F‐Flortaucipir tau PET and reduced CSF dopamine metabolites, representing a hypometabolic mismatch.^34^ Parallel to ^18^F‐FDG PET in DLB cohorts, PPMI studies indicate in sporadic and *LRRK2* PD that S+ patients have reduced DAT striatal uptake^11^ and some frontoparietal cortical atrophy, but with relative preservation of subcortical volume compared to S– patients.^41^ Therefore, we posit that patients with DLB or PD may all harbor varying profiles of mismatch between structural and molecular imaging that manifests in worse neurotransmission deficits in S+ patients and worse atrophy in S– patients.

Here, we aim to determine distinctive spatial patterns of dopaminergic deficit and subcortical volume loss based on α‐synuclein SAA status in PPMI participants with PD. We assessed ^123^I‐Ioflupane DAT SPECT and magnetic resonance imaging (MRI) data from motor‐impaired patients with sporadic PD and genetic PD associated with *LRRK2* and *GBA* variants. We hypothesize across sporadic, *LRRK2*, and *GBA* PD that S+ participants with PD will have more striatal DAT reduction than MRI atrophy, as α‐synuclein is tightly linked with dopaminergic denervation. Conversely, we predict that S– participants with PD will have fewer DAT uptake changes but more subcortical atrophy to account for their motor presentation, perhaps having concomitant AD neuropathologies associated with downstream neurodegeneration. We propose that DAT/MRI mismatch is linked to nonmotor symptoms such as impaired cognition or olfaction, depending on SAA status. Comparison of different imaging, clinical and CSF markers, allows us to investigate these predictions.

## Patients and Methods

### Patient Cohort

From the PPMI database (https://ppmi-info.org/), we included 728 participants with parkinsonism, including sporadic PD (n = 490), genetic PD with *LRRK2* variants (n = 158), and genetic PD with *GBA* variants (n = 80). Participants with sporadic PD were defined as patients with PD who did not have *LRRK2*, *GBA*, or other known familial PD variants. Participants with genetic PD related to *PRKN* (n = 14) and *SNCA* variants (n = 12) were not studied due to low frequency in this cohort. Patients with genetic PD harboring both *LRRK2* and *GBA* variants (n = 8) were also excluded due to small sample size and potentially overlapping biology. *LRRK2* exon variants included G2019S (n = 140), R1441G (n = 15), R1441C (n = 1), N1437H (n = 1), I2020T (n = 1). *GBA* variants included N409S (n = 66), L483P (n = 8), IVS2 + 1GA (n = 2), L29Afs*18 (n = 2), R159W (n = 1), R502C (n = 1). Baseline data were collated including sex, age, education, disease duration, Montreal Cognitive Assessment (MOCA), Movement Disorders Society–Unified PD Rating Scale (MDS‐UPDRS) Part III, and University of Pennsylvania Smell Identification Test (UPSIT) (accessed 4/2025). For exploratory analyses, we applied accepted definitions of cognitive impairment as MOCA<26 and hyposmia as UPSIT<34. See summary in Table 1.

**TABLE 1:** Clinical data for PPMI with cerebrospinal fluid (CSF) α‐synuclein seeding amplification assays (SAA) (S) markers

Diagnosis, gene, and SAA status	Sex (F/M)	Age (y)	Education (y)	Duration (y)	MDS‐UPDRS Part III	MOCA	UPSIT
Sporadic PD S– (n = 24)	8/16	67.0 (10.7)	15.7 (3.0)	0.6 (0.4)	21.5 (6.4)	27.3 (2.2)	34.2 (3.5)
Sporadic PD S+ (n = 466)	162/304	62.5 (9.3)	15.7 (2.8)	0.6 (0.6)	21.4 (9.0)	27.1 (2.4)	21.9 (7.7)
*LRRK2* PD S– (n = 57)	36/21	68.5 (6.5)	13.6 (4.7)	2.8 (2.1)	17.4 (7.4)	25.1 (3.3)	29.5 (6.6)
*LRRK2* PD S+ (n = 101)	41/60	60.5 (8.9)	16.1 (3.5)	2.9 (2.1)	20.1 (10.1)	26.8 (2.6)	23.0 (7.8)
*GBA* PD S– (n = 6)	2/4	63.2 (10.5)	16 (2.4)	1.6 (1.9)	22.3 (10.5)	26.7 (2.2)	31 (4.7)
*GBA* PD S+ (n = 74)	28/46	61.6 (10.1)	16.4 (3.3)	2.7 (2.4)	23.3 (12.2)	26.3 (2.6)	18 (7.1)

*Note*: Frequencies are shown for sex (F/M). Mean (with standard deviation) is shown for age, education, disease duration (all in years), Movement Disorders Society–Unified PD Rating Scale (MDS‐UPDRS) Part III, Montreal Cognitive Assessment (MOCA), and the University of Pennsylvania Smell Identification Test (UPSIT).

### CSF Biomarkers

CSF α‐synuclein SAA status (S+ vs. S–) was determined based on PPMI data (SAA_Biospecimen_Analysis_Results, accessed 2/2025). PPMI cases with S– status in this study were classified as not having type 1 or type 2 inclusions on CSF α‐synuclein SAA. Baseline CSF amyloid‐β_42_ (Aβ_42_) and pTau_181_ levels were obtained in 478 PPMI participants (Current_Biospecimen_Analysis_Results, accessed 3/2025). We assessed the level of pTau_181_ relative to Aβ_42_ in a ratio of pTau_181_/Aβ_42_, as a validated, sensitive measure of Alzheimer's pathology.^13^, ^42^

### Image Analysis and Striatal DAT/MRI Mismatch

Baseline imaging measures were obtained from the PPMI database for participants with ^123^I‐Ioflupane DAT SPECT scans (DaTScan_SBR_Analysis, accessed 4/2025) and MRI volumetric data (FS7_ASEG_VOL, accessed 4/2025). DAT scan data were preprocessed based on Chang 0 elliptical attenuation correction with site‐specific *μ* derived from phantoms. DAT‐specific binding ratios (SBR) were calculated from left and right putamen and caudate regions as (target count density/occipital count density) – 1. MRI volumetry from PPMI‐derived *FreeSurfer version 7.3.2* segmentations was evaluated for left and right putamen, caudate, nucleus accumbens, globus pallidus, thalamus, hippocampus, amygdala, cerebellar cortex, and cerebellar white matter, as well as the brainstem and total intracranial volume. Comparisons for MRI‐based measures were corrected for total intracranial volume, as per the Statistical Analysis section. See Supporting Information Figure 1S for subcortical brain atlas labels. Additional analyses compared the “lowest” or most affected side of bilateral structures with minimal DAT SBR or MRI volume per literature.^7^, ^11^ Similar to prior mismatch work,^31^, ^32^, ^33^ we assessed structure/function discordance between MRI atrophy and DAT scans via a DAT/MRI ratio defined as local (DAT SBR)/(MRI volume in cm^3^) for left and right caudate and putamen. 3D glass brain maps were rendered using MRIcroGL^43^ based on *χ* ^2^ statistics from likelihood ratio tests comparing imaging data in S+ versus S– patients with covariates of age, sex, education, disease duration (and total intracranial volume for MRI‐based measures). Color maps visualize lower measures in S+ than S– patients with blue, and lower measures in S– than S+ patients in red. See Supporting Information Table 1S for additional summary statistics.

**FIG. 1:**
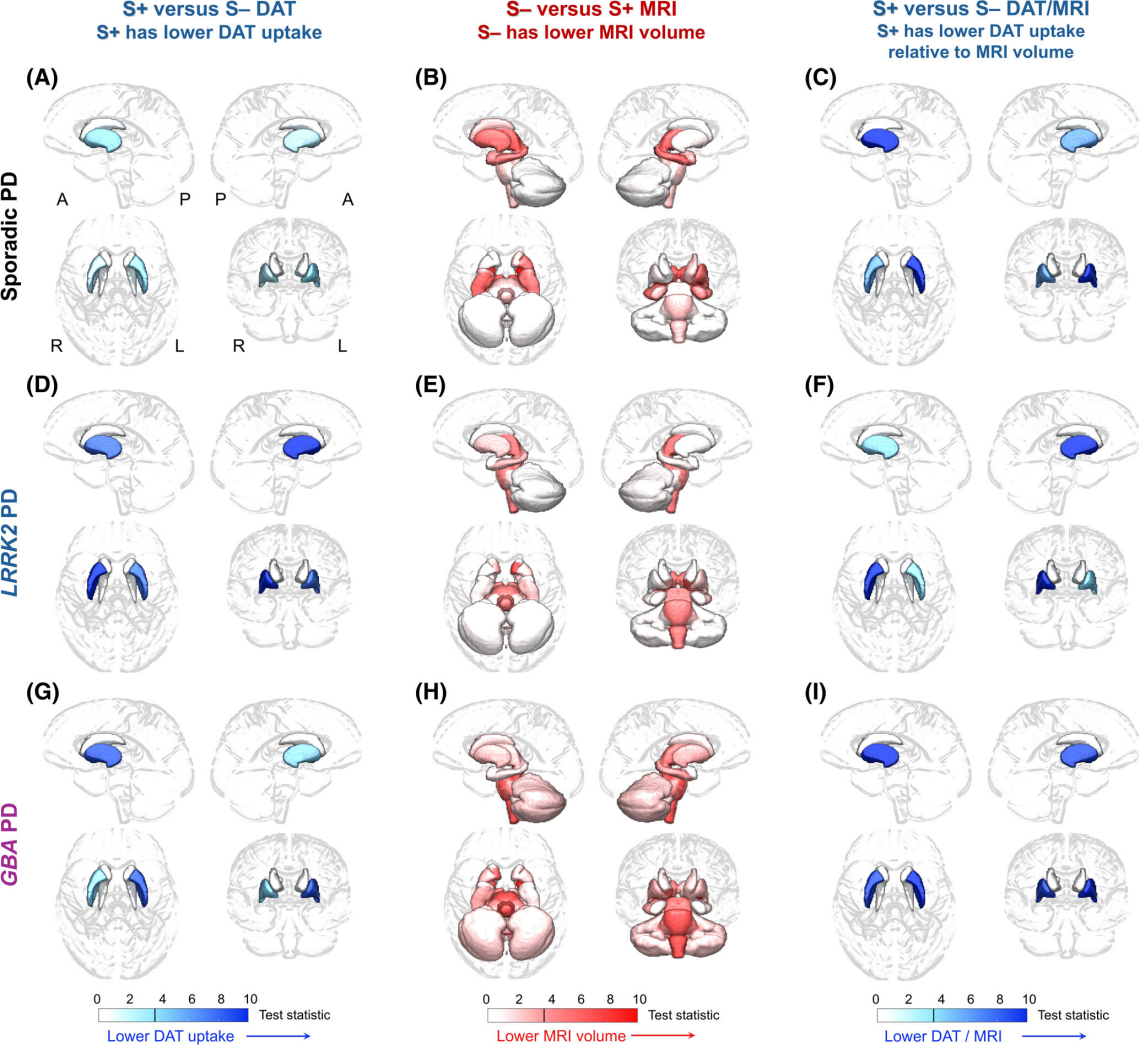
Imaging reveals a DAT/MRI mismatch signature in S+ versus S– participants with sporadic and genetic Parkinson's disease (PD). Regional 3D rendering glass brain maps in sporadic PD visualize (**A**) lower DAT SBR uptake in putamen of S+ patients compared to S– patients (blue), (**B**) reduced subcortical MRI volume in S– patients than S+ patients (red), and (**C**) worse putamen DAT/MRI ratios in S+ patients than S– patients (blue). Similar findings are observed for *LRRK2* PD with (**D**) DAT uptake, (**E**) MRI volume, and (**F**) DAT/MRI ratio, as well as for *GBA* PD with (**G**) DAT uptake, (**H**) MRI volume, and (**I**) DAT/MRI ratio. Color scale shows *χ* ^2^ test statistic from likelihood ratio tests with covariates of sex, education, age, disease duration (and intracranial volume for MRI measures). Blue colors show lower DAT, MRI, or DAT/MRI ratio measures in S+ than in S– patients. Red colors portray lower MRI volume in S– patients than S+ patients. [Color figure can be viewed at wileyonlinelibrary.com]

### Statistical Analysis

Statistical analysis was performed using *R version 4.1.2*. Statistical tests were two‐sided. Relative frequency comparisons were performed using Pearson *χ* ^2^ tests. All comparisons for CSF markers (such as the ratio of pTau_181_/Aβ_42_ levels) and image‐derived continuous variables (such as DAT SBR and MRI volumes) were performed with likelihood ratio tests by linear regression to adjust for covariates. Covariates included sex, age, and disease duration, along with total intracranial volume for MRI‐based measures. Additional covariates of dominant handedness and side of motor symptoms were included in models presented in the Supporting Information. For bilateral regions, except the brainstem, with significant group differences on both sides, *p*‐values are listed first for the left and then the right regions. Multiple‐comparison adjustment was performed using Benjamini–Hochberg correction with a false discovery rate of 0.05. Box plots show data points as dots, mean as X, median as middle box line, first quartile (Q1) and third quartile (Q3) as box edges (denoting inclusive interquartile range [IQR]), whiskers as minimum/maximum points, and outliers based on thresholds <Q1–1.5(IQR) or > Q3 + 1.5(IQR).

## Results

### In Sporadic PD, *LRRK2* PD, and *GBA* PD, S+ Participants Have Lower Putamen DAT Uptake, Whereas S– Participants Have Smaller Subcortical Volume

From the PPMI cohort of participants with PD and dopaminergic depletion on DAT scan, we assessed the discordance between DAT and MRI scans (DAT/MRI mismatch). First, we examined imaging differences in sporadic PD. S+ participants with sporadic PD had nonsignificant trends toward decreased left putamen DAT uptake compared to S– patients with sporadic PD (*p* = 0.059; Fig. 1A) after adjusting for covariates. Interestingly, S– patients with sporadic PD had significantly smaller volumes than S+ patients with sporadic PD in left putamen (*p* = 0.023), as well as the left and right globi pallidi (*p* = 0.001, *p* = 0.003, respectively), thalami (*p* = 0.011, *p* = 0.014), and hippocampi (*p* = 0.036, *p* = 0.019; Fig. 1B). Based on the differences in DAT versus MRI, we constructed the regional DAT/MRI ratio to compare DAT uptake changes relative to MRI atrophy. S+ sporadic PD patients harbored disproportionately worse left and right putaminal DAT uptake relative to MRI volume (*p* = 0.001, *p* = 0.017; Fig. 1C). Thus, S+ status is linked to lower putamen DAT uptake, whereas S– status is associated with worse subcortical volume in sporadic PD.

Next, we contrasted structural and molecular imaging in S+ versus S– participants with *LRRK2*‐associated PD. S+ patients with *LRRK2* PD had decreased left and right putaminal DAT uptake (*p* = 0.006, *p* < 0.001, respectively; Fig. 1D) compared to S– patients with *LRRK2* PD. Parallel to the findings of lower subcortical volumes in S– versus S+ patients with sporadic PD, S– patients with *LRRK2* PD patients also had smaller volume than their S+ counterparts in subcortical regions, including left nucleus accumbens (*p* = 0.010) and the left and right thalami (*p =* 0.035, *p* = 0.030) (Fig. 1E). With the DAT/MRI ratio, S+ patients with *LRRK2* PD had lower right putamen DAT uptake relative to volume (*p* < 0.001; Fig. 1F) than S– patients.

Furthermore, we assessed for dissociations in DAT and MRI scans in *GBA*‐associated PD. S+ participants with *GBA* PD had attenuated left putamen DAT uptake *p* = 0.004) (Fig. 1G) compared to S– *GBA* participants. Conversely, S– patients with *GBA* PD had reduced volumes than S+ *GBA* PD in right nucleus accumbens (*p* = 0.018), right thalamus (*p =* 0.029), and both left and right globi pallidi (*p* = 0.002, *p* = 0.008, respectively) (Fig. 1H). For the DAT/MRI ratio, S+ patients with *GBA* PD had lower left and right putaminal DAT uptake relative to MRI volume (*p* < 0.001, *p* = 0.003; Fig. 1I) than S– patients with *GBA* PD. Our findings indicate convergence of DAT/MRI dissociation by α‐synuclein SAA status across sporadic and genetic PD.

**FIG. 2:**
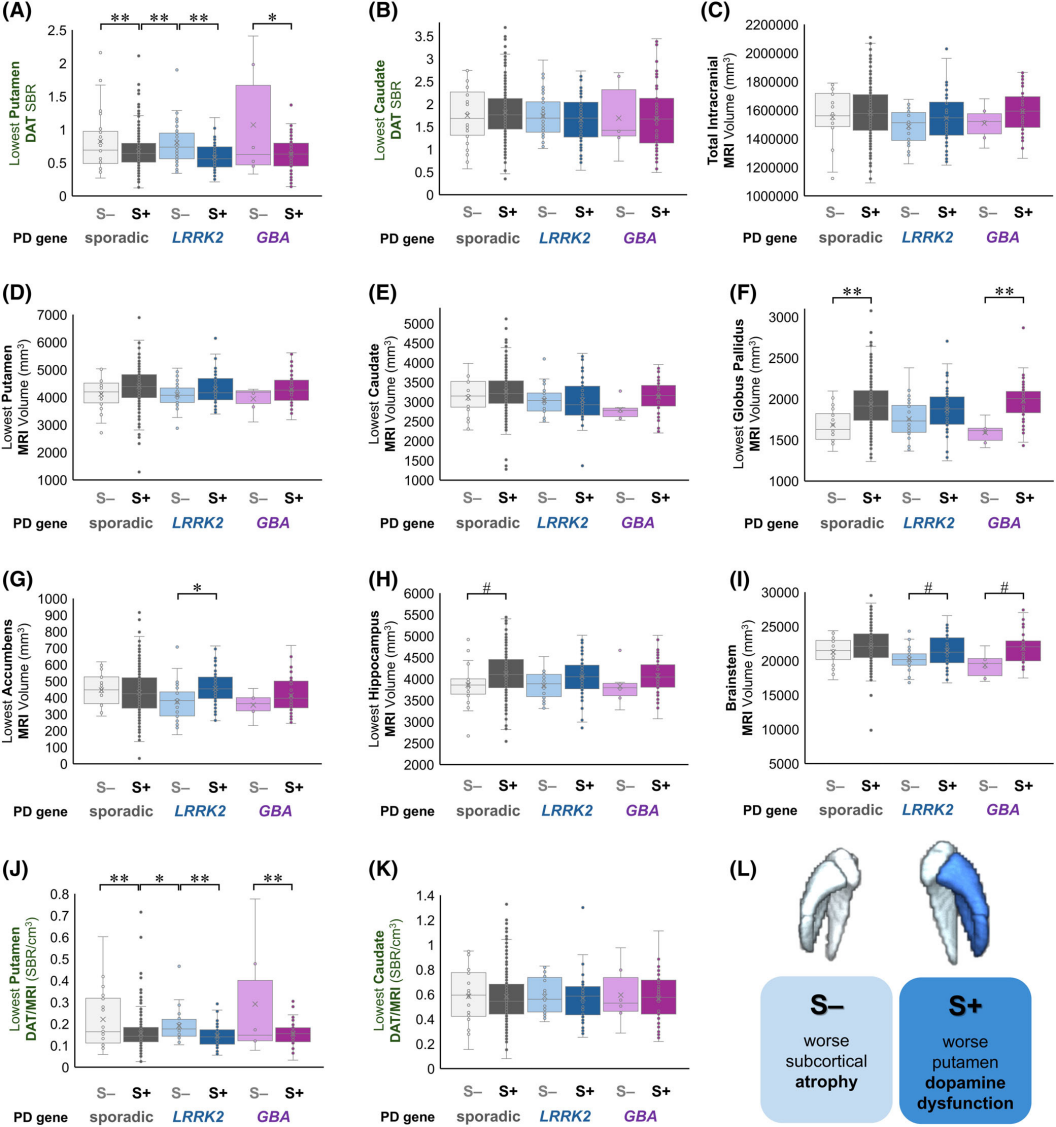
Most affected hemisphere subcortical regions have worse DAT uptake in S+ participants and lower MRI volume in S– participants in sporadic and genetic PD. DAT SBR uptake is shown for lowest (**A**) putamen and (**B**) caudate. Similar MRI volumes are observed for (**C**) total intracranial volume, lowest (**D**) putamen, and (**E**) caudate across groups, whereas (**F**) globus pallidus, (**G**) nucleus accumbens, (**H**) hippocampus, and (**I**) brainstem volumes show some group differences. DAT/MRI ratios (DAT uptake SBR/MRI volume in cm^3^) differ by S+ status in lowest (**J**) putamen but not (**K**) caudate. (**L**) S– patients have reduced subcortical volume, and S+ patients have dopaminergic denervation. Comparisons are made using likelihood ratio tests with covariates of age, sex, education, disease duration (and intracranial volume for MRI, except in panel **C**). #*p* < 0.05 before multiple‐comparison adjustment. *p < 0.05, **p < 0.005. [Color figure can be viewed at wileyonlinelibrary.com]

To further quantify these differences without laterality effects as per PD literature,^7^, ^11^ we calculated imaging measures based on the most affected or the “lowest” of the left and right values for bilateral regions of interest. Across PD gene status, S+ participants had worse putamen DAT SBR in the lower of the two hemispheres than S– participants for sporadic PD (*p* < 0.001), *LRRK2* (*p* = 0.004), and *GBA* PD (*p* = 0.007; Fig. 2A), accounting for covariates. Lowest caudate DAT SBR (Fig. 2B) and total intracranial MRI volume (Fig. 2C) did not significantly differ across PD groups. Lowest putamen (Fig. 2D) and caudate (Fig. 2E) volumes were fairly similar across sporadic and monogenic PD groups when comparing S+ and S– groups. However, volumes of other subcortical regions differed by α‐synuclein SAA status. Lowest globus pallidus volume was smaller in S– than S+ groups for sporadic PD (*p* < 0.001) and *GBA* PD (*p* = 0.001; Fig. 2F). Lowest nucleus accumbens volume was diminished in S– patients with *LRRK2* PD relative to S+ patients with *LRRK2* PD (*p =* 0.008; Fig. 2G). Lowest hippocampus and brainstem volumes were smaller in S– groups than S+ groups, but this did not survive multiple‐comparison adjustment (Fig. 2H,I). The DAT/MRI ratio accentuated this decoupling in dopaminergic degeneration and atrophy. Across PD gene status, lowest putamen DAT/MRI ratio was decreased more in S+ than S– groups, including sporadic (*p* < 0.001), *LRRK2* (*p* = 0.001), and *GBA* PD (*p* = 0.002; Fig. 2J). Significant changes were not seen for lowest caudate DAT/MRI ratios (Fig. 2K). Additionally, we applied covariates for dominant handedness and side of motor symptoms at the time of disease onset and found no substantial differences in the spatial pattern or magnitude of imaging findings (Supporting Information Fig. 2S), supporting the robustness of the observed discordance in DAT and MRI when comparing S+ and S– patients with PD. Cumulatively, we saw a dichotomy in phenotypes by α‐synuclein SAA status, with more pronounced atrophy in S– patients and more prominent dopaminergic deficit in S+ patients with PD (Fig. 2L).

### Cognitive Impairment and Hyposmia Are Associated with Worse DAT/MRI Mismatch in PD

We evaluated whether DAT/MRI mismatch correlated with nonmotor clinical features such as cognitive impairment (low MOCA<26) and hyposmia (low UPSIT<34). Because S+ status is associated with relative DAT deficit, whereas S– status is associated with relative MRI atrophy, we hypothesized that patients with worse nonmotor symptoms exhibit higher DAT/MRI ratio for S– status and lower DAT/MRI ratio for S+ status. In exploratory analyses limited by sample size, our findings supported this correspondence of nonmotor symptoms with relative atrophy in S– patients or DAT deficit in S+ patients. Cognitively impaired S– participants with sporadic PD had worse relative putamen atrophy (higher DAT/MRI ratio) than cognitively normal S– participants with sporadic PD (*p* = 0.049). Hyposmic S+ participants with *LRRK2* PD showed relative putamen DAT dysfunction (lower DAT/MRI ratio) compared to normosmic S+ participants with *LRRK2* PD (*p* = 0.039). Refer to Supporting Information Figure 3S. These results convey that cognitive impairment in S– patients is associated with worse MRI atrophy relative to DAT denervation, whereas hyposmia in S+ patients is associated with a worse DAT deficit relative to MRI volume.

**FIG. 3:**
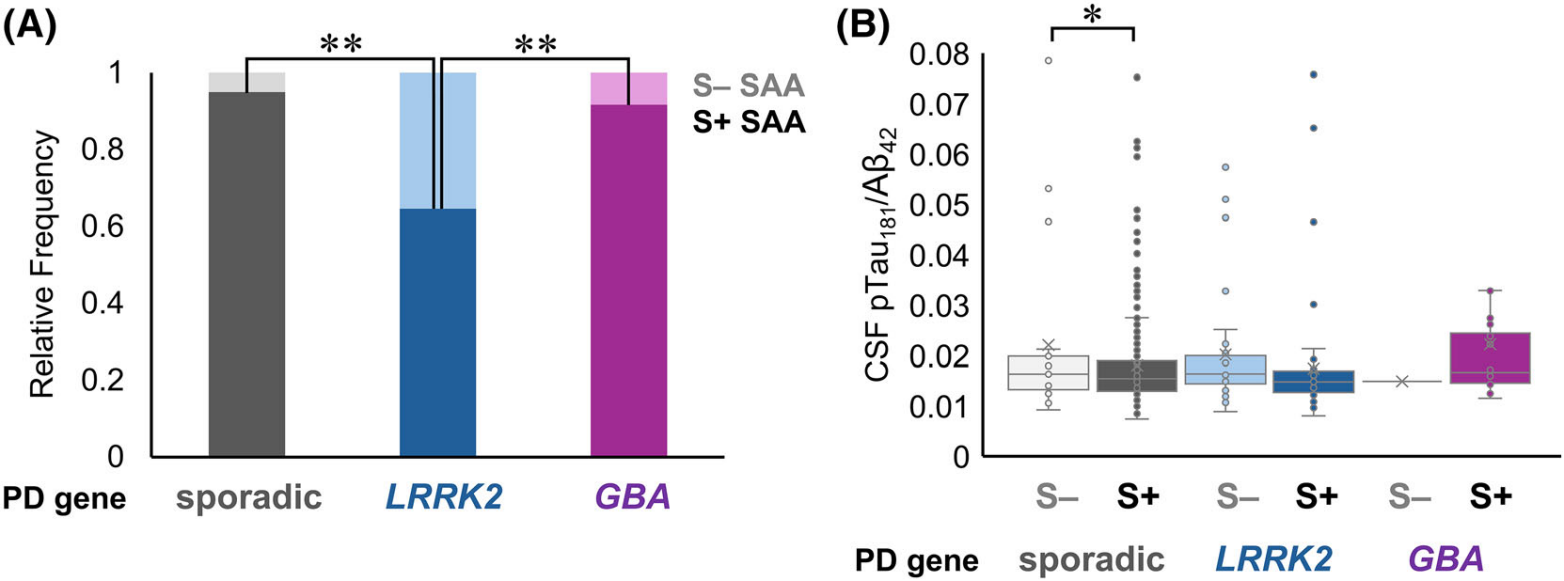
Cerebrospinal fluid (CSF) biomarkers of pathology and neurotransmission in sporadic and genetic Parkinson's disease (PD). (**A**) Plots show sporadic PD and *GBA* PD have >90% S+ status compared to 64% in *LRRK2* PD. Boxplots depict (**B**) CSF pTau_181_/Aβ_42_ ratio stratified by SAA in sporadic PD demonstrates that S– patients have higher pTau_181_/Aβ_42_ ratio than S+ patients. Comparisons were performed using likelihood ratio tests with covariates of age, sex, education, and disease duration. *p < 0.05, **p < 0.005. [Color figure can be viewed at wileyonlinelibrary.com]

### CSF Markers Related to AD Differ by α‐Synuclein Status in PD

Finally, we observed how CSF α‐synuclein SAA and AD measures vary among participants with sporadic PD, *LRRK2* PD, and *GBA* PD. We found 92% to 95% of participants with sporadic PD and *GBA* PD had S+ status, whereas *LRRK2* PD had less frequent S+ status (64%, *p* < 0.001; Table 1; Fig. 3A), which is substantiated by in vivo and ex vivo studies.^10^, ^11^, ^27^ S– participants with sporadic PD had similar covariate‐adjusted CSF levels of Aβ_42_ but higher pTau_181_/Aβ_42_ ratio than S+ participants with sporadic PD (*p =* 0.048; Fig. 3B). No significant differences were observed for these biomarkers in participants with genetic PD. Our result in sporadic PD is concordant with CSF data^44^ and autopsy studies^45^ of patients with clinically diagnosed PD, in which brains with nigrostriatal dopaminergic denervation without Lewy pathology were instead enriched for AD neuropathologic change. This raises the possibility of Alzheimer's copathology contributing to downstream motor impairment in those without Lewy pathology but clinically diagnosed with PD.

## Discussion

Determining α‐synuclein SAA status is essential to characterize the pathological substrate in patients with a PD clinical phenotype.^10^, ^11^ Although PET α‐synuclein radiotracers are in development,^46^, ^47^, ^48^, ^49^ the current CSF standard to determine α‐synuclein status in vivo is α‐synuclein SAA, which has confirmed postmortem findings by showing that the majority of patients with PD across PD gene status have S+ status in vivo.^2^, ^3^, ^4^, ^5^, ^6^, ^7^, ^8^, ^11^ However, SAA tests do not provide information on brain regional patterns of disease and may not be granular enough to finely characterize the heterogeneity of S+ versus S– status in various types of parkinsonism.

Therefore, we turned to MRI and DAT imaging to investigate spatial patterns of S+ versus S– status in 728 symptomatic PPMI participants with sporadic PD, *LRRK2* PD, and *GBA* PD. Intriguingly, we found across PD gene status that S+ status was associated with putaminal dopaminergic denervation on DAT SPECT, whereas S– status was related to subcortical volume loss in regions such as globus pallidus, nucleus accumbens, and thalamus. The putamen is an early hotspot of neurodegeneration in PD, before the involvement of the ipsilateral caudate and contralateral putamen,^50^ and our findings from baseline imaging data support this concept. Moreover, the predilection for subcortical volume loss in S– status is consistent with a recent PPMI study, noting subcortical preservation but cortical atrophy of frontoparietal regions in S+ patients over S– patients with PD,^41^ suggesting a discordance between cortical and subcortical neurodegenerative volume loss in PD across α‐synuclein status. Here, DAT uptake reductions in S+ patients were disproportionate to the degree of striatal volume loss relative to S– patients, revealing a DAT/MRI mismatch that is concordant with literature on metabolic and structural dissociation in LBD.^23^, ^31^ For instance, patients with mixed AD + DLB and dually positive amyloid‐β and α‐synuclein markers (A+S+) have greater parieto‐occipital mismatch between ^18^F‐FDG PET and MRI scans and lower CSF dopamine metabolites compared to A+S– cognitively impaired patients.^34^ Additional LBD studies revealed that changes in molecular rather than structural imaging are more readily seen in α‐synucleinopathy.^37^, ^38^, ^39^, ^40^ Therefore, the observed decoupling of DAT binding and MRI atrophy in α‐synucleinopathy is a reasonable finding that aligns with prior work describing dissociation of ^18^F‐FDG PET and MRI in LBD.

Across different PD gene statuses, studies portray how S– patients may have more cognitive impairment and S+ patients are more likely to have hyposmia. Moreover, patients with *LRRK2* PD are more likely to have S– status and may have worse cognitive impairment than their S+ counterparts, implying distinct pathophysiology.^7^, ^11^ Though exploratory, our study suggested that worse DAT/MRI mismatch corresponded to worse clinical symptoms. Cognitively impaired S– participants with sporadic PD had worse dopaminergic denervation relative to atrophy than those with normal cognition. Hyposmic S+ participants with *LRRK2* PD had worse volume loss relative to dopaminergic dysfunction than those with normal olfaction. Future studies can better assess the association of DAT/MRI mismatch with symptomatology in S– and S+ patients with PD.

In comparing CSF biomarkers, we saw >90% of patients with sporadic PD or *GBA* PD and ~ 64% of patients with *LRRK2* PD had S+ status, which is supported by literature.^2^, ^7^, ^11^, ^27^ Compared to the S+ group, S– patients with sporadic PD had elevated levels of CSF pTau_181_/Aβ_42_ ratio, a sensitive marker of AD (co)pathology.^42^ This finding is in line with in vivo studies showing associations between raised CSF and plasma pTau levels with AD copathology as measured by CSF Aβ_42_ and ^18^F‐RO948 tau PET in patients with PD.^44^ Moreover, postmortem data implicate higher Alzheimer's pathology with nigrostriatal dopaminergic loss in patients clinically diagnosed with PD but without Lewy body pathology at autopsy.^45^

Combining these threads of inquiry toward a rationale for different DAT/MRI mismatch profiles in S+ versus S– patients with PD, we postulated that α‐synuclein pathology impairs dopaminergic neuron integrity and function more so than structural volume changes in S+ patients with PD. This may stem from unique pathophysiological properties of misfolded α‐synuclein species that promote cellular dysfunction before precipitating overt death of dopaminergic neurons.^29^, ^30^, ^31^ Conversely, in the absence of Lewy pathology, parkinsonism could be driven more by structural damage to components of the dopaminergic circuit caused by non‐Lewy pathologies, such as vascular, AD, or TDP‐43 copathology,^45^ which may contribute to subcortical volume loss in S– patients but not to reductions in dopaminergic function to the same extent as in S+ patients. Notably, autopsy studies reveal that patients with PD without Lewy pathology often have abundant α‐synuclein oligomers (via proximity ligation assay) localizing to brainstem and hippocampus.^51^, ^52^ These findings of α‐synuclein oligomers enriched in subcortical regions of patients with PD without Lewy bodies (likely SAA negative) may be relevant to our results. Future studies with new biomarkers may substantiate or refute these hypotheses.

Knowledge gaps must be bridged to translate the basic pathobiology of α‐synuclein, *LRRK2*, and *GBA* with clinical diagnosis and management. *LRRK2* and *GBA* both encode proteins essential for endolysosomal recycling and autophagosomal degradation; mutations to both genes are implicated in downstream α‐synuclein accumulation.^9^, ^10^ Our studies were too underpowered to uncover the granularity of differential symptomatology or markers associated with each gene and SAA status, especially because S– status in sporadic or *GBA* PD is infrequent. Although we did not observe substantial differences in spatial DAT/MRI patterns, neurological function, or CSF pTau_181_/Aβ_42_ levels across all PD gene statuses, such distinctions may be detected in larger cohorts. Future investigations combining CSF and imaging markers of brain structure and function may illuminate pathophysiological and clinical correlates of *LRRK2* and *GBA*.

### Limitations and Future Directions

Our study has several limitations. The clinical cohort available is based on participants with a primarily motor phenotype in the PPMI study, such that the sample is more biased toward a predominantly PD‐like presentation and enriched for S+ status versus a general movement disorder population. Additional cohorts from community‐based samples, including more participants with mixed presentation or Parkinson‐plus syndromes such as MSA, progressive supranuclear palsy (PSP), or corticobasal degeneration (CBD), should be examined to validate our signatures of the DAT/MRI mismatch. Although we observe similar DAT/MRI mismatch relationships across sporadic PD, *LRRK2* PD, and *GBA* PD, substantial pathobiological differences underlie these distinct diseases. For example, S– status is much more common in *LRRK2* PD than *GBA* PD. Though we found similar patterns in DAT deficit and MRI atrophy across PD gene statuses, the *GBA* group had the lowest sample size and, therefore, the least power to detect differences in imaging and CSF markers when comparing S+ versus S– groups. Despite this smaller sample size, we still found differences within the *GBA* group, lending support to the generality of DAT/MRI mismatch. Future studies will better characterize DAT/MRI mismatch in larger cohorts of sporadic PD, *LRRK2* PD, and *GBA* PD over time, though longitudinal imaging data are less available. CSF α‐synuclein SAA also has drawbacks. Several SAA tests exhibit different sensitivities for α‐synuclein pathology based on different stages, such as worse detection rates in patients with lower α‐synuclein load or olfactory involvement.^7^, ^20^ Some early S+ patients may have been misclassified with S– status, which might influence assessments. Comparing performance in detecting PD versus MSA, various SAA tests may perform worse for MSA.^13^, ^17^, ^18^ Although the PPMI cases with S– status here were classified as not having type 1 (PD) or type 2 (MSA) inclusions, it is possible that MSA pathology or four‐repeat tauopathy could contribute to disease in some S– cases diagnosed with sporadic PD.^53^ Because glial α‐synuclein inclusions also occur in LBD,^54^ follow‐up investigations could determine how markers of neuronal versus nonneuronal α‐synuclein relate to DAT/MRI mismatch. Quantitative kinetic measures for α‐synuclein SAA are being studied,^55^, ^56^, ^57^ but not always available in clinical cohorts, so extending this work to compare SAA kinetic profiles with neuroimaging could be fruitful. SAA parameters may differ for *LRRK2* PD,^58^ which warrants further study. Macroscopic volumetric measures may not fully capture microscopic structural changes, and future work may enumerate the relationship between DAT scans and diffusion tractography, susceptibility‐weighted, and neuromelanin‐sensitive MRI sequences.^59^ Alzheimer's pathology markers are not as widely available in PPMI, and other studies may quantify amyloid‐β and tau pathology using PET.

Overall, we demonstrate that S+ status on CSF α‐synuclein SAA is associated with more severe DAT scan reduction, whereas S– status is linked to smaller subcortical volume across sporadic PD and genetic PD associated with *LRRK2* and *GBA* variants. S– patients with sporadic PD may also have higher CSF pTau_181_/Aβ_42_ ratios, suggesting that they could display possible downstream effects of Alzheimer's pathology. Cognitive and olfactory impairment may correspond to DAT/MRI discordance depending on α‐synuclein SAA. Our findings lend credence to the hypothesis that α‐synuclein pathology attenuates functional neuronal integrity more than structural volume and carry potential to improve our understanding of biomarkers, pathophysiology, and clinical symptoms of α‐synucleinopathy and PD.

## Author Roles

(1) Research project: A. Conception and design, B. Organization, C. Execution; (2) Statistical analysis: A. Design, B. Execution, C. Review and critique; (3) Manuscript preparation: A. Writing of the first draft, B. Review and critique.

M.T.D.: 1A, 1B, 1C, 2A, 2B, 2C, 3A, 3B

S.R.D.: 1A, 2C, 3A, 3B

P.K.: 2C, 3A, 3B

J.A.V.: 2C, 3A, 3B

Y.L.: 3B

L.X.: 3B

P.Y.: 3B

L.M.S.: 3B

J.A.D.: 3B

A.S.: 1A, 2C, 3A, 3B

D.A.W.: 1A, 2C, 3A, 3B

I.M.N.: 1A, 2C, 3A, 3B

## Financial Disclosures of All Authors (for the Past 12 Months)

Funding was provided by the National Institute on Aging (NIA) via a Ruth L. Kirschstein National Research Service Award (NIA F30 AG074524 and T32‐NS091006‐10), a research project grant (NIA R01 AG072796), a University of Pennsylvania Alzheimer's Disease Research Center grant (NIA P30 AG072979), and Institute for Translational Medicine and Therapeutics pilot grant (UL1‐TR001878), as well as The Michael J. Fox Foundation. The authors thank lab members for constructive discussions and the Parkinson's Progression Markers Initiative (PPMI) investigators, staff, participants, and families. This manuscript is dedicated to the families of the authors, as well as the patients and families from PPMI. PPMI is a public–private partnership funded by The Michael J. Fox Foundation for Parkinson's Research and funding partners, including 4D Pharma, AbbVie, AcureX, Allergan, Amathus Therapeutics, Aligning Science Across Parkinson's, AskBio, Avid Radiopharmaceuticals, Bial Portela, BioArctic, Biogen, Biohaven, BioLegend, BlueRock Therapeutics, Bristol‐Myers Squibb, Calico Labs, Capsida Biotherapeutics, Celgene, Cerevel Therapeutics, Coave Therapeutics, DaCapo Brainscience, Denali, Edmond J. Safra Foundation, Eli Lilly, Gain Therapeutics, General Electric HealthCare, Genentech, GlaxoSmithKline (GSK), Golub Capital, Handl Therapeutics, Insitro, Jazz Pharmaceuticals, Johnson & Johnson Innovative Medicine, Lundbeck, Merck, Meso Scale Discovery, Mission Therapeutics, Neurocrine Biosciences, Neuron23, Neuropore Therapies, Pfizer, Piramal, Prevail Therapeutics, Roche, Sanofi, Servier, Sun Pharma Advanced Research Company, Takeda, Teva, Union Chimique Belge (UCB), Vanqua Bio, Verily, Voyager Therapeutics, the Weston Family Foundation, and Yumanity Therapeutics. This work was supported in part by the Intramural Research Program of the NIA and the Center for Alzheimer's and Related Dementias (CARD) under award number AG000534.

## Financial Disclosures and Conflicts of Interest

S.R.D. reports grants/fees from Nia Therapeutics and Rancho Biosciences outside this work. L.X. reports fees from Galileo CDS, Inc., outside this work. L.X. has become an employee of Siemens Healthineers, but his work on this study was conducted during his employment at the University of Pennsylvania. J.A.V. reports honoraria/fees from the International Parkinson and Movement Disorder Society and the University City Science Center. L.M.S. reports grants/fees from Biogen, Diadem, Fujirebio, Roche Diagnostics, and Siemens, all outside this work. A.S. reports grants/fees from Bial, Merck, and Parkinson Study Group outside this work. D.A.W. reports grants/fees from Beckman Coulter, Biogen, Eli Lilly, Functional Neuromodulation, GE Healthcare, GSK, and Qynapse outside this work. I.M.N. reports grants/fees from Biogen and Eisai outside this work. There are no remaining disclosures to report. Author disclosures are available in the Supporting Information.

## Supporting information


**Figure S1.** Atlas labels for magnetic resonance imaging (MRI) subcortical regions.
**Figure S2.** DAT/MRI imaging mismatch with additional covariates of dominant handedness and side of motor symptoms. Regional 3D rendering glass brain maps in sporadic Parkinson's disease (PD) visualize (**A**) lower DAT SBR uptake in putamen of S+ patients compared to S– patients (blue), (**B**) reduced subcortical MRI volume in S– patients than S+ patients (red), and (**C**) worse putamen DAT/MRI ratios in S+ patients than S– patients (blue). Similar findings are seen for *LRRK2* PD with (**D**) DAT uptake, (**E**) MRI volume, and (**F**) DAT/MRI ratio, as well as for *GBA* PD with (**G**) DAT uptake, (**H**) MRI volume, and (**I**) DAT/MRI ratio. Color scale shows *χ* ^2^ test statistic from likelihood ratio tests with covariates of dominant handedness, dominant side of motor symptoms at time of disease onset, sex, education, age, disease duration (and intracranial volume for MRI measures). Blue colors show lower DAT, MRI, or DAT/MRI ratio measures in S+ than S– patients. Red colors portray lower MRI volume in S– patients than S+ patients.
**Table S1.** Summary statistics for imaging measures across groups.

**Data S1.** Supporting information.

## Data Availability

Data used in the preparation of this article were obtained from the Parkinson's Progression Markers Initiative (PPMI) database (https://ppmi-info.org/access-data-specimens/download-data). All data, including raw and processed scans, clinical and biomarker spreadsheets, and data dictionaries, are free and publicly available at the PPMI data archive (https://ida.loni.usc.edu/login.jsp?project=PPMI). Additional documents, including study protocols and methods, are available online. Data access can be requested on the website.

## Appendix A

### A.1 Parkinson's Progression Marker Initiative Authors

**PPMI Steering Committee**:

Kenneth Marek, MD^1^ (Principal Investigator); Shirley Lasch, MBA^1^; Caroline Tanner, MD, PhD^2^ (Site Investigator); Tanya Simuni, MD^3^ (Site Investigator); Christopher Coffey, PhD^4^ (Statistics Core, PI); Karl Kieburtz, MD, MPH^5^ (Clinical Core, PI); Renee Wilson^5^; Brit Mollenhauer, MD^6^ (Bioanalytics Core, co‐PI; Site Investigator); Douglas Galasko, MD^7^ (Bioanalytics Core, co‐PI; Site Investigator); Tatiana Foroud, PhD^8^ (Genetics Coordination Core and Biorepository, PI); Lana Chahine, MD^9^ (Site Investigator); Andrew Siderowf, MD, MSCE^9^; John Seibyl, MD (Imaging Core, PI)^1^; Arthur Toga, PhD^10^ (Bioinformatics Core, PI); Andrew Singleton, PhD^11^ (Genetics Core, PI); Daniel Weintraub, MD^9^ (Cognitive and Behavioral); John Trojanowski, MD, PhD^9^; Leslie Shaw, PhD^9^; Duygu Tosun‐Turgut, PhD^2^ (DTI, PI); Kathleen Poston, MD, MS (fMRI, PI)^15^; Susan Bressman, MD^27^; Kalpana M. Merchant, MD^54^; Werner Poewe, MD^12^ (Site Investigator); Todd Sherer, PhD^13^; Sohini Chowdhury^13^; Mark Frasier, PhD^13^; Catherine Kopil, PhD^13^; Anna Naito, PhD^13^; Vanessa Arnedo.^13^

**PPMI Study Cores (**additional members): *Clinical Coordination Core*: Ray Dorsey, PhD^5^; Cynthia Casaceli, MBA^5^; Imaging Core: Nichole Daegele^1^; Justin Albani^1^ Statistics Core: Chelsea Caspell‐Garcia, MS ^4^; Liz Uribe, MS^4^; Eric Foster ^4^; Jeff Long, PhD^4^; Nick Seedorff^4^; *Bioinformatics Core*: Karen Crawford, MLIS^10^; *BioRepository*: Danielle Elise Smith^8^; Paola Casalin^14^; Giulia Malferrari^14^; *Genetics Coordination and Pathology Core*: Cheryl Halter^8^; Laura Heathers.^8^

**PPMI Site Investigators**: David Russell, MD, PhD^1^; Stewart Factor, DO^16^; Penelope Hogarth, MD^17^; David Standaert, MD, PhD^18^; Amy Amara, MD, PhD^18^; Robert Hauser, MD, MBA^19^; Joseph Jankovic, MD^20^; Matthew Stern, MD^9^; Shu‐Ching Hu, MD PhD^21^; Gretchen Todd^21^; Rachel Saunders‐Pullman MD^27^; Irene Richard, MD^23^; Marie H Saint‐Hilaire, MD^22^; Klaus Seppi, MD^12^; Holly Shill, MD^24^; Hubert Fernandez, MD^25^; Claudia Trenkwalder, MD^6^; Wolfgang Oertel MD^42^; Daniela Berg, MD^26^; Kathrin Brockman, MD^26^; Isabel Wurster MD^26^; Liana Rosenthal, MD^28^; Yen Tai, MD^29^; Nicola Pavese, MD^29^; Paolo Barone, MD, PhD^30^; Stuart Isaacson, MD^31^; Alberto Espay, MD, MSc^32^; Dominic Rowe, MD, PhD^33^; Melanie Brandabur MD^35^; James Tetrud MD^35^; Grace Liang MD^35^; Alex Iranzo, MD^34^; Eduardo Tolosa MD^34^; Karen Marder, MD^36^; Maria de Arriba Sanchez, MD^37^; Leonidis Stefanis, MD, PhD^38^; Maria Jose Marti, MD, PhD^34^; Javier Ruiz Martinez, MD, PhD^37^; Jean‐Christophe Corvol, MD^39^; Jan O Assly, MD^40^; Salima Brillman, MD^35^; Nir Giladi, MD.^41^

**PPMI Coordinators**: Debra Smejdir^1^; Julia Pelaggi^1^; Farah Kausar, PhD^2^; Linda Rees, MPH^35^; Barbara Sommerfield, MSN, RN^16^; Madeline Cresswell^17^; Courtney Blair, MA^18^; Karen Williams^3^; Grace Zimmerman^5^; Stephanie Guthrie, MSN^18^; Ashlee Rawlins^18^; Leigh Donharl^19^; Christine Hunter, RN^20^; Baochan Tran^9^; Abigail Darin^9^; Carly Linder^9^; Marne Baca^21^; Heli Venkov^21^; Cathi‐Ann Thomas, RN, MS^22^; Raymond James, RN^22^; Beatrice Heim, MD^12^; Paul Deritis^23^; Fabienne Sprenger, MD^12^; Deborah Raymond^27^; Diana Willeke^6^; Zoran Obradov, CRC^24^; Jennifer Mule^25^; Nancy Monahan^25^; Katharina Gauss^26^; Deborah Fontaine, BSN, MS^7^; Daniel Szpak^7^; Arita McCoy^28^; Becky Dunlop^28^; Laura Marie Payne^29^; Susan Ainscough^30^; Lisbeth Carvajal^31^; Rebecca Silverstein^31^; Kristy Espay^32^; Madelaine Ranola^33^; Elisabet Mondragon Rezola^37^; Helen Mejia Santana^36^; Maria Stamelou, MD, PhD^38^; Alicia Garrido, MD^34^; Stephanie Carvalho, MS^39^; Anne Grete Kristiansen^40^; Krista Specketer^21^; Anat Mirlman.^41^

**ISAB** (Industry Scientific Advisory Board): Maurizio Facheris, MD^43^; Holly Soares, PhD^43^; Mark A. Mintun, MD^44^; Jesse Cedarbaum, MD^45^; Peggy Taylor, ScD^46^; Danna Jennings, MD^48^; Lawrence Slieker, PhD^48^; Brian McBride, PhD^49^; Colin Watson, PhD^49^; Etienne Montagut, MBA^49^; Zulfiqar Haider Sheikh^49^; Baris Bingol, PhD^50^; Remi Forrat^51^; Pablo Sardi, PhD^51^; Tanya Fischer, MD, PhD^51^; Alastair D. Reith, PhD^52^; Jan Egebjerg, PhD^53^; Lone Frydelund Larsen^53^; Nathalie Breysse, PhD^53^; Didier Meulien, MD^53^; Barbara Saba, MD^54^; Vera Kiyasova, MD, PhD^54^; Chris Min, MD, PhD^55^; Thomas McAvoy, PhD^55^; Robert Umek, PhD^56^; Philip Iredale, PhD^57^; Jeremy Edgerton, PhD^57^; Susan De Santi, PhD^58^; Christian Czech, PhD^59^; Frank Boess, PhD^59^; Jeffrey Sevigny, MD^59^; Thomas Kremer, PhD^59^; Igor Grachev, MD, PhD^60^; Kaplana Merchant, PhD^61^; Andreja Avbersek, MD^62^; Pierandrea Muglia, MD^62^; Alexandra Stewart, MBA^63^; Rene Prashad, PhD^63^, Johannes Taucher, MD^64^.

^1^Institute for Neurodegenerative Disorders, New Haven, CT

^2^University of California, San Francisco, CA

^3^Northwestern University, Chicago, IL

^4^University of Iowa, Iowa City, IA

^5^Clinical Trials Coordination Center, University of Rochester, Rochester, NY

^6^Paracelsus‐Elena Klinik, Kassel, Germany

^7^University of California, San Diego, CA

^8^Indiana University, Indianapolis, IN

^9^University of Pennsylvania, Philadelphia, PA

^10^Laboratory of Neuroimaging (LONI), University of Southern California, Los Angeles, CA

^11^National Institute on Aging, NIH, Bethesda, MD

^12^Innsbruck Medical University, Innsbruck, Austria

^13^The Michael J. Fox Foundation for Parkinson's Research, New York, NY

^14^BioRep Milan, Italy

^15^Stanford University Medical Center, Stanford, CA

^16^Emory University of Medicine, Atlanta, GA

^17^Oregon Health and Science University, Portland, OR

^18^ University of Alabama at Birmingham, Birmingham, AL

^19^University of South Florida, Tampa, FL

^20^Baylor College of Medicine, Houston, TX

^21^University of Washington/University of Washington and VA Puget Sound Health, Seattle, WA

^22^Boston University, Boston, MA

^23^University of Rochester, Rochester, NY

^24^Banner Research Institute, Sun City, AZ

^25^Cleveland Clinic, Cleveland, OH

^26^University of Tübingen, Tübingen, Germany

^27^Beth Israel Medical Center, New York, NY

^28^Johns Hopkins University, Baltimore, MD

^29^Imperial College of London, London, UK

^30^University of Salerno, Salerno, Italy

^31^Parkinson's Disease and Movement Disorders Center, Boca Raton, FL

^32^University of Cincinnati, Cincinnati, OH

^33^Macquarie University, Sydney Australia

^34^Hospital Clinic of Barcelona, Barcelona, Spain

^35^The Parkinson's Institute, Sunnyvale, CA

^36^Columbia University Medical Center, New York, NY

^37^Hospital Donista, San Sebastian, Spain

^38^Foundation for Biomedical Research of the Academy of Athens, Athens, Greece

^39^Hospital Pitie‐Salpetriere, Paris, France

^40^St Olav's Hospital, Norway

^41^Tel Aviv Sourasky Medical Center, Tel Aviv, Israel

^42^Philipps University Marburg, Germany

^44^Avid Radiopharmaceuticals, Inc, Philadelphia, PA

^48^Eli Lilly and Company, New York, NY

^43^Abbvie, Chicago, IL

^45^Biogen Idec, Cambridge, MA

^46^BioLegend, San Diego, CA

^47^Bristol‐Myers Squibb Company, New York, NY

^49^GE Healthcare, Little Chalfont, United Kingdom

^50^Genentech Inc., South San Francisco, CA

^51^Genyzme Sanofi, Cambridge, MA

^52^GlaxoSmithKline Pharmaceuticals R&D, Brentford, United Kingdom

^53^H. Lundbeck A/S Copenhagen, Denmark

^54^Institut de Recherches Internationales Servier, Croissy, France

^55^Merck, Kenilworth, NJ

^56^Meso Scale Discovery Rockville, MD

^57^Pfizer Inc, Cambridge, MA

^58^Piramal Life Sciences, Berlin, Germany

^59^Roche, Basel, Switzerland

^60^Teva, Petah Tekva, Israel

^61^TransThera Consulting Co., Portland, OR

^62^UCB Pharma S.A., Brussels, Belgium

^63^Weston Brain Institute, Toronto, ON

^64^Takeda, Osaka, Japan
