# Supplementary material for: Discordance of Dopaminergic Dysfunction and Subcortical Atrophy by α‐Synuclein Status in Sporadic and Genetic Parkinson's Disease
Source: Mov Disord. 2026 Jan 28;41(5):1101–13. doi: 10.1002/mds.70186 (PMC13206170; doi:10.1002/mds.70186)
Supplement: Supplementary file 1 — Figure S1. Atlas labels for magnetic resonance imaging (MRI) subcortical regions. Figure S2. DAT/MRI imaging mismatch with additional covariates of dominant handedness and side of motor symptoms. Regional 3D rendering glass brain maps in sporadic Parkinson's disease (PD) visualize (A) lower DAT SBR uptake in putamen of S+ patients compared to S– patients (blue), (B) reduced subcortical MRI volume in S– patients than S+ patients (red), and (C) worse putamen DAT/MRI ratios in S+ patients than S– patients (blue). Similar findings are seen for LRRK2 PD with (D) DAT uptake, (E) MRI volume, and (F) DAT/MRI ratio, as well as for GBA PD with (G) DAT uptake, (H) MRI volume, and (I) DAT/MRI ratio. Color scale shows χ 2 test statistic from likelihood ratio tests with covariates of dominant handedness, dominant side of motor symptoms at time of disease onset, sex, education, age, disease duration (and intracranial volume for MRI measures). Blue colors show lower DAT, MRI, or DAT/MRI ratio measures in S+ than S– patients. Red colors portray lower MRI volume in S– patients than S+ patients. Table S1. Summary statistics for imaging measures across groups. [file MDS-41-1101-s002.docx]

**Supporting Information**


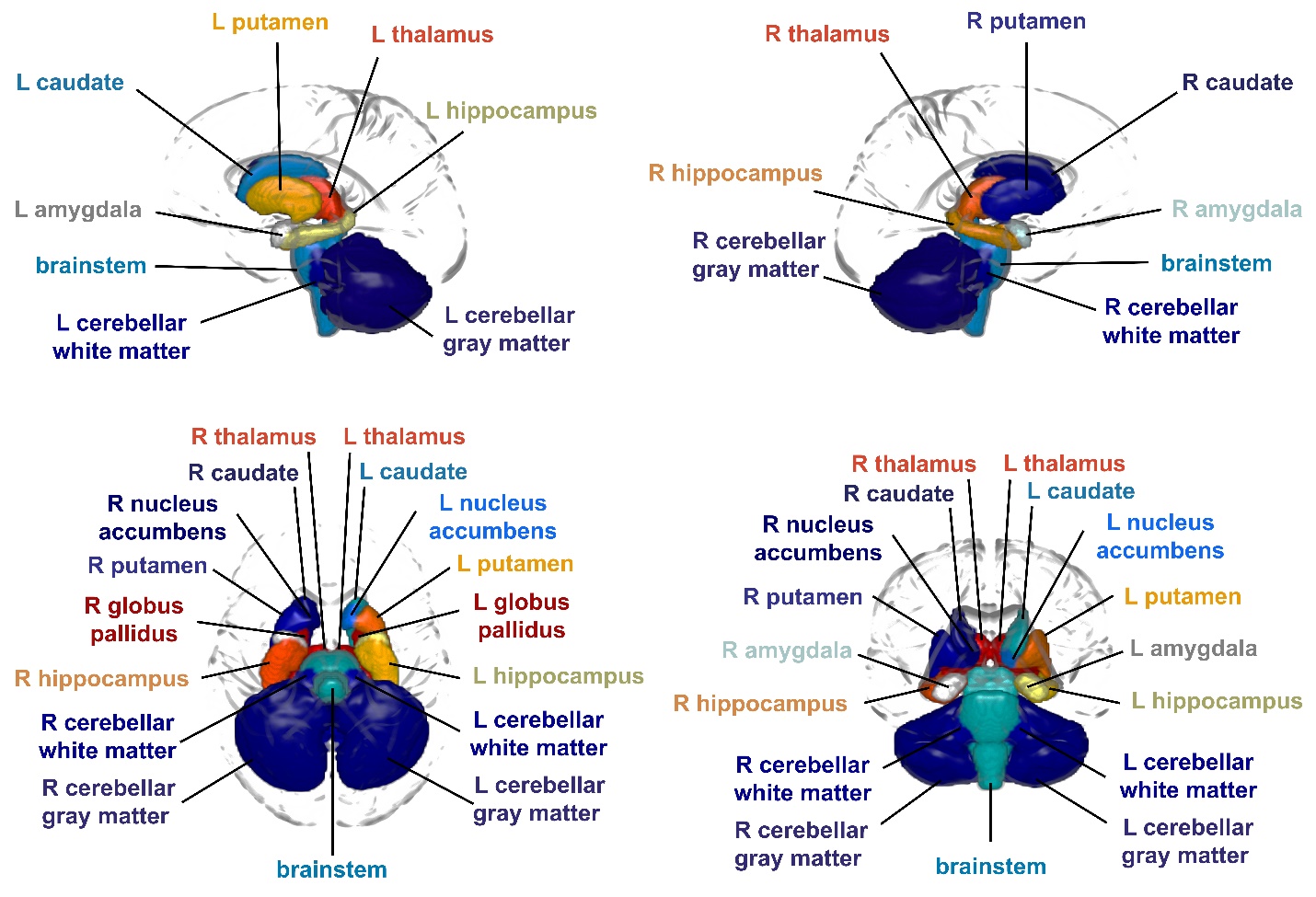


**FIG. 1S. *Atlas labels for MRI subcortical regions.***


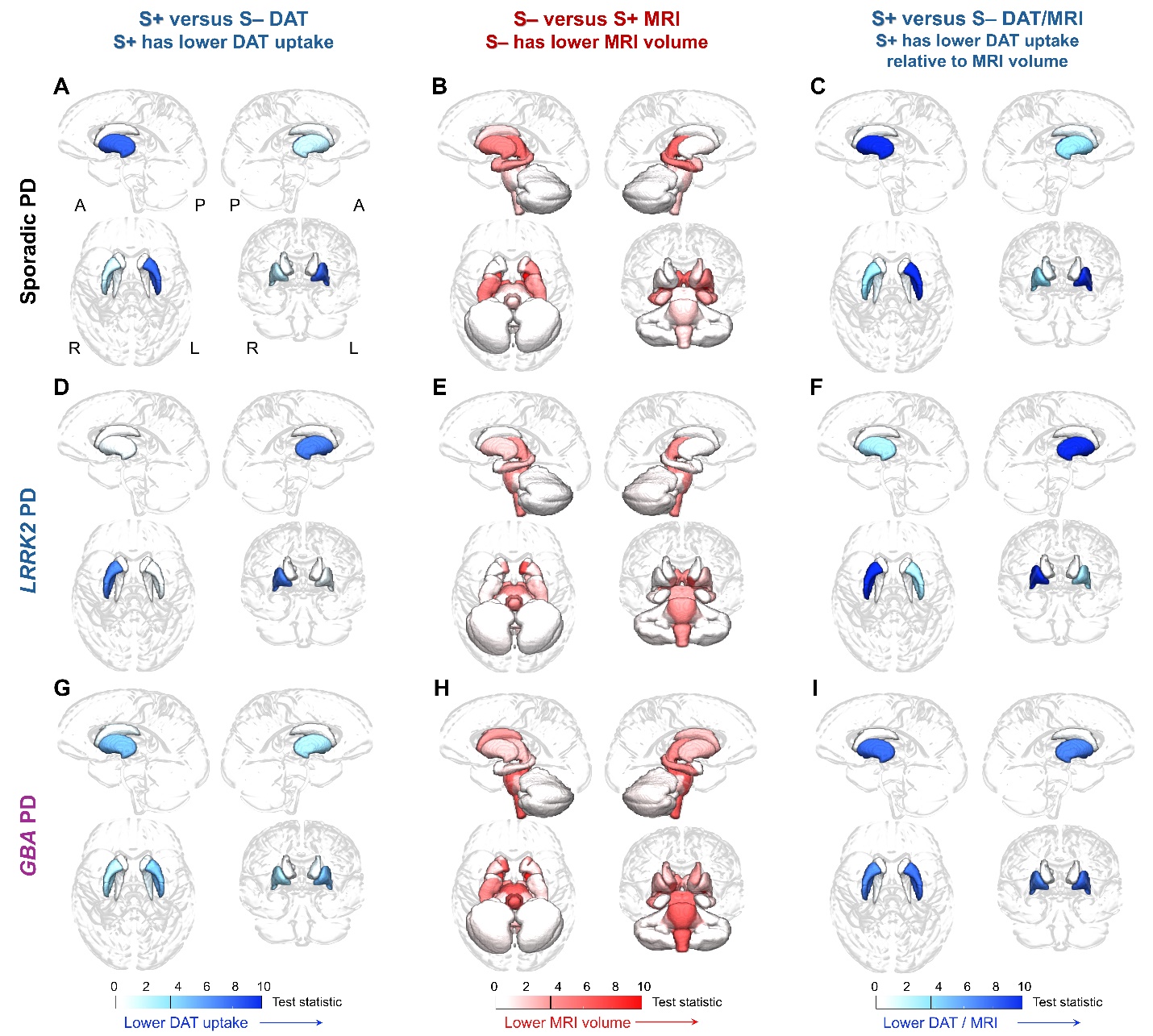


**FIG. 2S**. **DAT/MRI imaging mismatch with additional covariates of dominant handedness and side of motor symptoms**. Regional 3D rendering glass brain maps in sporadic PD visualize (**A**) lower DAT SBR uptake in putamen of S+ patients compared to S– patients (blue), (**B**) reduced subcortical MRI volume in S– patients than S+ patients (red) and (**C**) worse putamen DAT/MRI ratios in S+ patients than S– patients (blue). Similar findings are seen for *LRRK2* PD with (**D**) DAT uptake, (**E**) MRI volume and (**F**) DAT/MRI ratio, as well as for *GBA* PD with (**G**) DAT uptake, (**H**) MRI volume and (**I**) DAT/MRI ratio. Color scale shows *χ*^2^ test statistic from likelihood ratio tests with covariates of dominant handedness, dominant side of motor symptoms at time of disease onset, sex, education, age, disease duration (and intracranial volume for MRI measures). Blue colors show lower DAT, MRI or DAT/MRI ratio measures in S+ than. S– patients. Red colors portray lower MRI volume in S– patients than S+ patients.


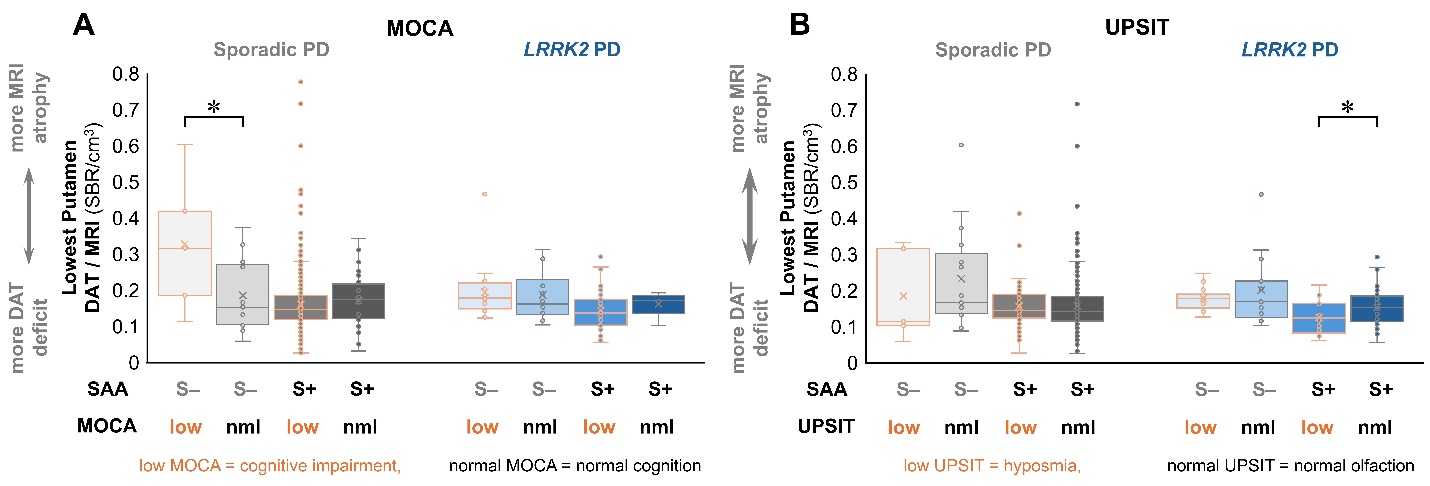


**FIG. 3S. Association of impaired cognition and olfaction with differential DAT/MRI mismatch ratio.** (**A**) Lowest putamen DAT/MRI ratio (lower is more DAT deficit relative to atrophy, higher is more atrophy relative to DAT deficit) in patients with sporadic and *LRRK2* PD with or without cognitive impairment on the Montreal Cognitive Assessment (low MOCA<26 is impaired vs. MOCA ≥26 is normal). (**B**) Lowest putamen DAT/MRI ratio in patients with sporadic and *LRRK2* PD with or without hyposmia on the University of Pennsylvania Smell Identification Test (low UPSIT<34 is hyposmic vs. UPSIT≥34 is normal). Comparisons are by likelihood ratio tests with covariates of age, sex, education, disease duration and intracranial volume for MRI. **P*<0.05.

**TABLE 1S. *Summary statistics for imaging measures across groups.*** Mean (standard deviation) shown for DAT SBR, MRI volume (mm^3^) and DAT/MRI ratio (SBR/cm^3^) in sporadic PD, *LRRK2* PD, *GBA* PD groups as stratified by CSF α-synuclein SAA status.

| **Brain Region** | **Sporadic PD S–** | **Sporadic PD S+** | ***LRRK2* PD S–** | ***LRRK2* PD S+** | ***GBA* PD S–** | ***GBA* PD S+** |
| --- | --- | --- | --- | --- | --- | --- |
| **DAT uptake**  (SBR) |  |  |  |  |  |  |
| left putamen | 0.96 (0.59) | 0.82 (0.36) | 0.89 (0.35) | 0.72 (0.29) | 1.13 (0.92) | 0.73 (0.28) |
| right putamen | 0.96 (0.55) | 0.85 (0.34) | 0.94 (0.44) | 0.67 (0.25) | 1.08 (0.90) | 0.74 (0.36) |
| left caudate | 1.91 (0.72) | 2.01 (0.60) | 1.91 (0.48) | 1.83 (0.56) | 1.75 (0.77) | 1.88 (0.75) |
| right caudate | 1.91 (0.72) | 2.00 (0.60) | 1.87 (0.56) | 1.80 (0.60) | 1.80 (0.80) | 1.85 (0.77) |
| **MRI volume**  (mm^3^) |  |  |  |  |  |  |
| intracranial volume | 1551681  (194979) | 1579922  (179446) | 1487055  (1543751) | 1543751  (178478) | 1509237  (124603) | 1591558  (148631) |
| left putamen | 4144 (613) | 4480 (666) | 4100 (480) | 4344 (583) | 3965 (495) | 4319 (529) |
| right putamen | 4411 (581) | 4542 (665) | 4304 (504) | 4442 (587) | 4027 (424) | 4431 (526) |
| left caudate | 3136 (503) | 3317 (506) | 3082 (402) | 3110 (537) | 2799 (266) | 3162 (458) |
| right caudate | 3392 (510) | 3396 (561) | 3159 (398) | 3209 (596) | 2962 (285) | 3262 (442) |
| left globus pallidus | 1725 (233) | 2026 (280) | 1811 (244) | 1949 (281) | 1689 (178) | 2072 (248) |
| right globus pallidus | 1796 (212) | 1994 (308) | 1798 (231) | 1910 (280) | 1620 (190) | 2017 (287) |
| left nucleus accumbens | 470 (124) | 439 (142) | 380 (116) | 467 (120) | 363 (92) | 416 (108) |
| right nucleus accumbens | 519 (111) | 533 (124) | 465 (105) | 535 (114) | 393 (90) | 520 (107) |
| left thalamus | 6731 (944) | 7347 (1065) | 6585 (639) | 7102 (935) | 6314 (609) | 7206 (1035) |
| right thalamus | 6687 (922) | 7229 (955) | 6471 (522) | 6924 (862) | 6113 (839) | 7084 (881) |
| left hippocampus | 3858 (501) | 4117 (499) | 3848 (328) | 3942 (480) | 3835 (467) | 4071 (437) |
| right hippocampus | 3923 (617) | 4215 (503) | 4062 (429) | 4136 (488) | 3803 (366) | 4201 (464) |
| left amygdala | 1481 (341) | 1618 (287) | 1483 (279) | 1547 (284) | 1510 (280) | 1582 (257) |
| right amygdala | 1646 (320) | 1758 (261) | 1623 (306) | 1704 (244) | 1667 (303) | 1732 (245) |
| left cerebellar cortex | 52184 (5807) | 53033 (6865) | 49057 (5655) | 50115 (4656) | 49389 (5655) | 52190 (4656) |
| right cerebellar cortex | 53182 (6151) | 54005 (6677) | 49646 (5724) | 51054 (5237) | 49982 (3901) | 53391 (6714) |
| left cerebellar white matter | 14998 (2217) | 15704 (3003) | 14463 (2118) | 15451 (2536) | 13342 (1704) | 15666 (2573) |
| right cerebellar white matter | 14482 (1734) | 15011 (2554) | 13809 (1969) | 14770 (2243) | 13289 (1313) | 14863 (1870) |
| brainstem | 21290 (2105) | 22179 (2496) | 20220 (1779) | 21476 (2298) | 19368 (1953) | 21844 (2269) |
| **DAT/MRI** (SBR/cm^3^) |  |  |  |  |  |  |
| left putamen | 0.25 (0.17) | 0.19 (0.08) | 0.21 (0.08) | 0.18 (0.08) | 0.30 (0.28) | 0.18 (0.07) |
| right putamen | 0.24 (0.15) | 0.19 (0.09) | 0.21 (0.09) | 0.16 (0.06) | 0.28 (0.27) | 0.18 (0.08) |
| left caudate | 0.64 (0.25) | 0.63 (0.22) | 0.63 (0.15) | 0.61 (0.17) | 0.62 (0.25) | 0.62 (0.19) |
| right caudate | 0.60 (0.24) | 0.62 (0.23) | 0.61 (0.18) | 0.59 (0.22) | 0.61 (0.26) | 0.60 (0.21) |
