# Supplementary material for: Discordance of Dopaminergic Dysfunction and Subcortical Atrophy by α‐Synuclein Status in Sporadic and Genetic Parkinson's Disease
Source: Mov Disord. 2026 Jan 28;41(5):1101–13. doi: 10.1002/mds.70186 (PMC13206170; doi:10.1002/mds.70186)
Supplement: Supplementary file 2 — Data S1. Supporting information. [file MDS-41-1101-s001.docx]

| ICMJE DISCLOSURE FORM | |
| --- | --- |
| **Date:** | 10/10/2025 |
| **Your Name:** | Michael Tran Duong, Sandhitsu R. Das, Pulkit Khandelwal, Joaquin A. Vizcarra, Yue Li, Long Xie, Paul A. Yushkevich, Leslie M. Shaw, Jacob Dubroff, Andrew Siderowf, David A. Wolk, Ilya M. Nasrallah |
| **Manuscript Title:** | Discordance of dopaminergic dysfunction and subcortical atrophy by α-synuclein status in sporadic and genetic Parkinson Disease |
| **Manuscript Number (if known):** | MDS-25-1013 |
| In the interest of transparency, we ask you to disclose all relationships/activities/interests listed below that are related to the content of your manuscript. “Related” means any relation with for-profit or not-for-profit third parties whose interests may be affected by the content of the manuscript. Disclosure represents a commitment to transparency and does not necessarily indicate a bias. If you are in doubt about whether to list a relationship/activity/interest, it is preferable that you do so.  The author’s relationships/activities/interests should be defined broadly. For example, if your manuscript pertains to the epidemiology of hypertension, you should declare all relationships with manufacturers of antihypertensive medication, even if that medication is not mentioned in the manuscript.  In item #1 below, report all support for the work reported in this manuscript without time limit. For all other items, the time frame for disclosure is the past 36 months. | |

|  | | | **Name all entities with whom you have this relationship or indicate none (add rows as needed)** | **Specifications/Comments (e.g., if payments were made to you or to your institution)** |
| --- | --- | --- | --- | --- |
| **Time frame: Since the initial planning of the work** | | | | |
| **1** | All support for the present manuscript (e.g., funding, provision of study materials, medical writing, article processing charges, etc.)  **No time limit for this item.** | | \|  \| **None** \| \| --- \| --- \|  \| Ruth L. Kirschstein National Research Service Awards (NIA F30 AG074524 and T32-NS091006‐10) \| University of Pennsylvania Institute for Translational Medicine and Therapeutics pilot grant (UL1-TR001878) \| \| --- \| --- \| \| Research project grant (NIA R01 AG072796) \| Michael J. Fox Foundation \| \| University of Pennsylvania Alzheimer’s Disease Core Center grant (NIA P30 AG072979) \| Click the tab key to add additional rows. \| | |
| **Time frame: past 36 months** | | | | |
| **2** | | Grants or contracts from any entity (if not indicated in item #1 above). | \|  \| **None** \| \| --- \| --- \|  \|  \|  \| \| --- \| --- \| \|  \|  \| \|  \|  \| | |
| **3** | | Royalties or licenses | \|  \| **None** \| \| --- \| --- \|  \|  \|  \| \| --- \| --- \| \|  \|  \| \|  \|  \| | |
| **4** | | Consulting fees | \|  \| **None** \| \| --- \| --- \|  \| S.R.D. reports fees from Nia Therapeutics and Rancho Biosciences outside this work. \| A.D.S. reports fees from Bial, Merck and Parkinson Study Group outside this work. \| \| --- \| --- \| \| L.X. reports fees from Galileo CDS, Inc. L.X. has become an employee of Siemens Healthineers but his work on this study was conducted during his employment at the University of Pennsylvania. \| D.A.W. reports fees from Beckman Coulter, Biogen, Eli Lilly, Functional Neuromodulation, GE Healthcare, GSK and Qynapse outside of this work. \| \| J.A.V. reports honoraria/fees from the International Parkinson and Movement Disorder Society and the University City Science Center. \| I.M.N. reports fees from Biogen and Eisai outside this work. \| \| L.M.S. reports fees from Biogen, Diadem, Fujirebio, Roche Diagnostics and Siemens, all outside this work. \|  \| | |
| **5** | | Payment or honoraria for lectures, presentations, speakers bureaus, manuscript writing or educational events | \|  \| **None** \| \| --- \| --- \|  \| J.A.V. reports honoraria/fees from the International Parkinson and Movement Disorder Society and the University City Science Center. \|  \| \| --- \| --- \| \|  \|  \| \|  \|  \| | |
| **6** | | Payment for expert testimony | \|  \| **None** \| \| --- \| --- \|  \|  \|  \| \| --- \| --- \| \|  \|  \| \|  \|  \| | |
| **7** | | Support for attending meetings and/or travel | \|  \| **None** \| \| --- \| --- \|  \|  \|  \| \| --- \| --- \| \|  \|  \| \|  \|  \| | |
| **8** | | Patents planned, issued or pending | \|  \| **None** \| \| --- \| --- \|  \|  \|  \| \| --- \| --- \| \|  \|  \| \|  \|  \| | |
| **9** | | Participation on a Data Safety Monitoring Board or Advisory Board | \|  \| **None** \| \| --- \| --- \|  \|  \|  \| \| --- \| --- \| \|  \|  \| \|  \|  \| | |
| **10** | | Leadership or fiduciary role in other board, society, committee or advocacy group, paid or unpaid | \|  \| **None** \| \| --- \| --- \|  \|  \|  \| \| --- \| --- \| \|  \|  \| \|  \|  \| | |
| **11** | | Stock or stock options | \|  \| **None** \| \| --- \| --- \|  \|  \|  \| \| --- \| --- \| \|  \|  \| \|  \|  \| | |
| **12** | | Receipt of equipment, materials, drugs, medical writing, gifts or other services | \|  \| **None** \| \| --- \| --- \|  \|  \|  \| \| --- \| --- \| \|  \|  \| \|  \|  \| | |
| **13** | | Other financial or non-financial interests | \|  \| **None** \| \| --- \| --- \|  \|  \|  \| \| --- \| --- \| \|  \|  \| \|  \|  \| | |
|  | |  |  | |
| **Please place an “X” next to the following statement to indicate your agreement:** | | | | |
|  | | I certify that I have answered every question and have not altered the wording of any of the questions on this form. | | |
